# Polyaniline-Derived Nitrogen-Containing Carbon Nanostructures with Different Morphologies as Anode Modifier in Microbial Fuel Cells

**DOI:** 10.3390/ijms231911230

**Published:** 2022-09-23

**Authors:** Irina Lascu, Claudiu Locovei, Corina Bradu, Cristina Gheorghiu, Ana Maria Tanase, Anca Dumitru

**Affiliations:** 1 Faculty of Biology, University of Bucharest, Splaiul Independenței 91–95, 050095 Bucharest, Romania; 2 Faculty of Physics, University of Bucharest, P.O. Box MG-11, 077125 Magurele, Romania; 3 National Institute of Materials Physics, Atomistilor 405A, 077125 Magurele, Romania; 4 Extreme Light Infrastructure-Nuclear Physics (ELI-NP), “Horia Hulubei” National Institute for R&D in Physics and Nuclear Engineering, P.O. Box MG-6, 077125 Magurele, Romania

**Keywords:** microbial fuel cell, anode modification, polyaniline, carbonization, nitrogen-containing carbon nanostructures, biofilm, microbial diversity, extracellular electron transfer

## Abstract

Anode modification with carbon nanomaterials is an important strategy for the improvement of microbial fuel cell (MFC) performance. The presence of nitrogen in the carbon network, introduced as active nitrogen functional groups, is considered beneficial for anode modification. In this aim, nitrogen-containing carbon nanostructures (NCNs) with different morphologies were obtained via carbonization of polyaniline and were further investigated as anode modifiers in MFCs. The present study investigates the influence of NCN morphology on the changes in the anodic microbial community and MFC performance. Results show that the nanofibrillar morphology of NCNs is beneficial for the improvement of MFC performance, with a maximum power density of 40.4 mW/m^2^, 1.25 times higher than the anode modified with carbonized polyaniline with granular morphology and 2.15 times higher than MFC using the carbon cloth-anode. The nanofibrillar morphology, due to the well-defined individual nanofibers separated by microgaps and micropores and a better organization of the carbon network, leads to a larger specific surface area and higher conductivity, which can allow more efficient substrate transport and better bacterial colonization with greater relative abundances of *Geobacter* and *Thermoanaerobacter*, justifying the improvement of MFC performance.

## 1. Introduction

Bioelectrochemical systems (BES) represent different biofilm-based bioreactors that include microbial fuel cells (MFC), microbial electrolysis cells (MEC), microbial desalination cells (MDC), and microbial reverse electrodialysis cells (MRC) and hold great promise for sustainable production of energy and positive wastewater treatment [1,2,3,4,5,6]. Among them, microbial fuel cell (MFC) is an emerging technology that uses bacterial metabolism and electrochemical processes for both wastewater treatment and bioenergy production. MFC integrates bioelectrochemically active microorganism growth in the form of biofilms on the electrode surface to produce bioelectricity from a variety of organic substrates [1,7]. Anode materials, structure, and configuration, together with microbial biofilms colonizing anodic electrodes, are the key factors that affect the performance of MFCs [7,8,9,10,11,12]. The vital role of anode materials is given by their direct contact with bacteria, affecting bacterial attachments and the electron transfer rate from electrochemically active bacteria to the anode surface [13,14]. The main properties of the anode materials that can improve the MFC performance include electrical conductivity, chemical stability and durability, biocompatibility, cost and accessibility. A wide variety of materials, such as conventional and advanced carbon materials, metal-based or metal composites, conducting polymers, and composite materials, have been used to enhance the performance of MFCs [9,14]. Among them, carbon-based electrodes have been extensively studied due to their chemical stability and high electrical conductivity, but the hydrophobic characteristic of their surface does not favor bacterial adhesion and, as a consequence, electron transfer capacity. Thus, the use of MFC anodes modified with nanomaterials seems to be a promising strategy for the improvement of MFCs performance compared with plain carbon anodes [7,9]. In this aim, anode modification with carbon nanostructures increases the surface area for the attachment of microorganisms, which favors the kinetics of electron transfer [9,14]. As well, the incorporation of heteroatoms in the graphitic network can enhance both the conductivity and the catalytic activity of the material, attracting great interest in energy and environmental applications [15,16,17]. As reported recently, the incorporation of heteroatoms into carbon can disturb the sp^2^-hybridized graphitic network, interfering in charge distribution, and could tailor its electron-donor properties for required electrical and chemical performance, thus expanding the property of carbon materials [18,19]. To this extent, nitrogen-doped carbon nanostructures, such as nitrogen-doped ordered mesoporous carbon [20], N-doped carbon nanotube [21], nitrogen-doped graphene [22], self-nitrogen-doped carbon nanosheets [23], and nitrogen-doped carbon dots [24], were used for anode modification and show an improved MFC performance, which was attributed to the nitrogen functionality. Among various strategies that have been developed to introduce nitrogen functionality on the anode surface, direct annealing of N-containing precursors (i.e., polyaniline, polypyrrole, polydopamine) under an inert atmosphere [15,25,26,27,28] can simplify the preparation processes and reduce cost. Polyaniline (PANI), as a nitrogen-containing carbon precursor, has been widely investigated for electrochemical energy conversion and storage applications due to its easy synthesis, high environmental stability, low production cost, and high nitrogen content [19,27,28]. There are few studies that mention the use of carbonized PANI in BES [19,29,30,31]. Thus, carbonised PANI was used for the modification of a commercial sponge in order to improve the MFC performance [19], a graphene oxide-supported carbon nanofiber-like network derived from polyaniline was used as an anode in a glucose/O_2_ fuel cell [29], a novel three-dimensional (3D) carbon composite of PANI_1600_@CNTs was applied to enhance a enzymatic biofuel cell [30] and a 3D carbon foam with surface-anchored nitrogen-containing carbon nanoparticles derived from PANI as a freestanding anode in MFC [31].

Furthermore, the use of mixed microbial communities in MFCs (such as municipal wastewater, activated sludge and soil sediment) gained a lot of attention due to their stability and high power production compared with pure cultures [32,33]. Understanding the microbial consortium changes during the process would allow researchers to harness microbial functions to obtain more efficient electricity from MFCs.

In our study, we synthesized polyaniline with different morphologies, which were then used as precursors for nitrogen-containing carbon nanostructures (NCNs). The resulting NCNs with different morphology were used as anode modifiers in an MFC system loaded with municipal wastewater, and the microbial diversity of the resulting biofilms formed on the anode surface was analyzed, along with that of the initial wastewater, through high-throughput short read sequencing. The present study investigates how the changes in NCN morphology and anodic microbial community affect MFC performance, which, to our knowledge, has not been reported in the existing literature.

## 2. Results and Discussions

### 2.1. Characterization of Polymer Precursors and Corresponding Nitrogen-Containing Carbon Nanostructures

#### 2.1.1. Surface Characterization and Elemental Composition by SEM/EDX

**Figure 1:**
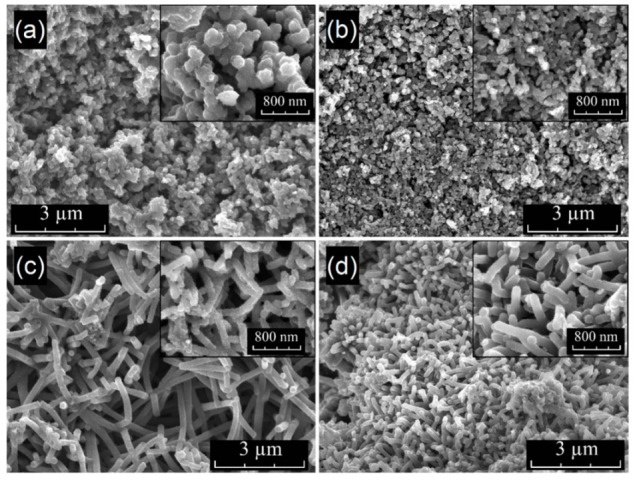
SEM images of (**a**) PANI-R (scale bar 3 μm), (**b**) PANI-R-900 (scale bar 3 μm), (**c**) PANI-T (scale bar 3 μm), and (**d**) PANI-T-900 (scale bar 3 μm). The insets present SEM images acquired at higher magnification with a scale bar of 800 nm.

Two different synthesis methods used in our work allowed for the synthesis of polyaniline with different morphologies, one with near-granular-shaped nanoparticles (PANI-R) and another with nanofibrillar morphology (PANI-T). Direct carbonization of polymeric precursors (PANI-R and PANI-T) allowed us to obtain NCNs with different morphologies (PANI-R-900 and PANI-T-900). The SEM micrographs depicted in Figure 1 of PANI-R and PANI-T samples clearly show two very distinct morphologies. In Figure 1a, the SEM images of PANI-R taken at different magnifications show the near granular shape of the nanoparticles, with a mean diameter of aggregated particles of approximately 150 nm. SEM images of PANI-R-900 (Figure 1b) indicate that the general morphological features are preserved after carbonization, with a sizable decrease in grain size. Figure 1c highlights the nanofiber morphology of the PANI-T sample with a rough surface of nanofibers, which are interconnected in bunch-like structures. In this case, the approximate mean diameter of nanofibers is around 130 nm, but some thicker rods may be observed because of the agglomeration of nanofibers. The carbonization process caused almost negligible changes in the morphology of PANI-T (Figure 1d); nonetheless, the PANI-T-900 sample presented a relatively smooth surface of nanofibers compared with PANI-T.

From energy dispersive X-ray analysis (EDX), the elemental compositions of all samples are summarized in Table 1, and the original data of all samples are presented in the Supplementary Materials (Figures S1–S4), which indicate the presence of C, N, O, and S atoms for PANI-R and PANI-T, with a higher at % of N and O in the case of PANI-R. The N content in PANI-R is close to its theoretical content calculated upon the chemical structure of the aniline unit as the atomic C:N ratio is 6 and is higher in the case of PANI-T. After carbonization of PANI-R and PANI-T, the EDX analysis (Table 1) indicates the presence of C, N and O atoms in both samples with an increased contribution of the carbon content and a significant drop in at % of oxygen, which is a common characteristic given by the carbonization process of the polymer precursors. Furthermore, the results show that a significant amount of nitrogen is preserved in the samples after carbonization, highlighting the high stability of the N-doped carbon nanostructures, which can enhance the conductivity and hydrophilicity of the graphitic network. The EDS mapping of PANI-R-900 and PANI-T-900 show that the N and O are homogeneously distributed in the graphitic network for both carbonized samples (Figure S5).

Thus, in our work, we synthesized polyaniline with different morphologies, which was further used as a polymeric precursor for NCNs. The results prove that the direct carbonization of polyaniline nanostructures is an easy and economical process to obtain nitrogen-rich carbon materials, preserving the general features of the precursor’s morphology, with a smaller decrease in nanoparticle size, as can be seen in the SEM images and EDX analysis of the polymer precursors and their corresponding NCNs.

#### 2.1.2. FT-IR Spectroscopy

**Figure 2:**
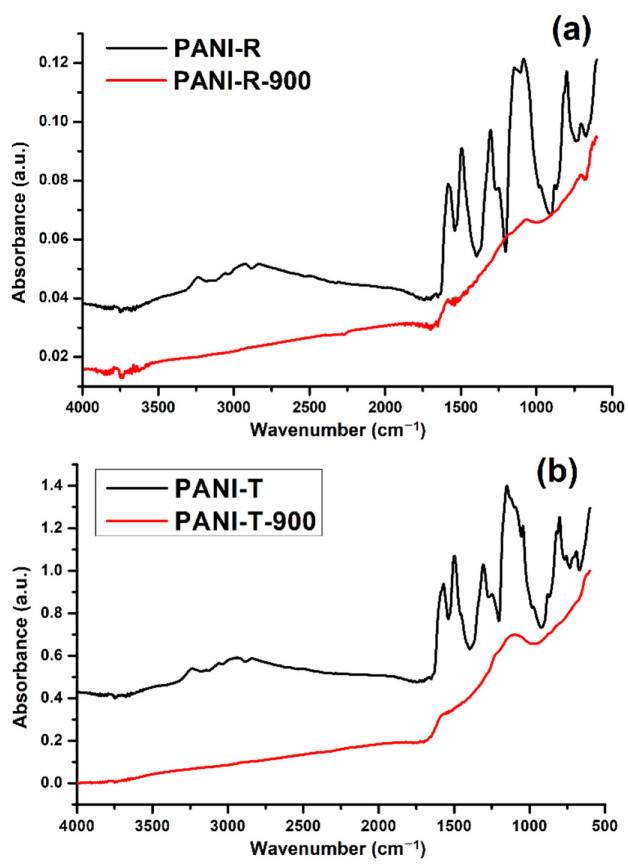
FTIR spectra of (**a**) PANI-R and PANI-R-900 and (**b**) PANI-T and PANI-T-900 samples.

In Figure 2, the FT-IR spectra of PANI-R and PANI-T are shown, along with their corresponding NCNs. The characteristic peaks of PANI-R (Figure 2a) appearing around ~1583 cm^−1^ and ~1493 cm^−1^ are assigned to the C=C stretching vibration of the quinoid and benzenoid rings, respectively. The band at ~1306 cm^−1^ can be attributed to the C–N–C stretching vibration or to the π-electron delocalization induced in the polymer through protonation, while the band at 1248 cm^−1^ is attributed to the C–N^+●^ stretching mode of the polaron structure, which is characteristic of the conducting protonated form of PANI [34,35,36]. The band around 1144 cm^−1^ can be assigned to a C-H bending vibration or the vibrations of the charged polymer units Q=NH^+^–B or B–NH^+●^–B (Q denotes the quinoid ring and B denotes the benzenoid ring), formed during protonation [34,35,37].

The bands at about 1080 cm^−1^ and 700 cm^−1^ correspond to hydrogen sulfate counterions, which are characteristic of the conducting form of PANI [35]. The bands appearing between 880 cm^−1^–800 cm^−1^ are attributed to the aromatic C–H vibrations. In addition to the above peaks, the spectrum of the PANI-R exhibits bands at 3238 cm^−1^ that are attributed to N-H stretching mode, and the absorption between ~3063 cm^−1^–2830 cm^−1^ corresponds to an aromatic C–H stretching vibration [34,37,38]. In comparison with the spectrum of PANI-R, the spectrum of PANI-T (Figure 2b) contains new additional bands positioned at 1448 cm^−1^, 1042 cm^−1^, 750 cm^−1^ and 688 cm^−1^, which can be associated with the different morphology of the samples, as reported previously [35]. Thus, the band at 1448 cm^−1^ corresponds to the skeletal C=C stretching vibration of the aromatic ring; 1042 cm^−1^ is due to the sulphonate groups attached to the aromatic rings [39]. The bands observed at 750 cm^−1^ and 688 cm^−1^ are related to the monosubstituted aromatic rings as terminal units or indicate more pronounced branching chains in the nanofiber samples [35,39,40].

The FTIR spectra of both carbonized samples show almost no peaks in the whole range of 400–4000 cm^−1^, except for the two broad bands with maxima at 1580 cm^−1^ and 1132 cm^−1^. The first one is assigned to the aromatic ring vibration mixed with C=N stretching vibration, and the second ones correspond to C–C and C–N vibrations, respectively. These bands are characteristic of carbon-like materials that have Raman G- and D-bands, which are not IR active. The presence of nitrogen in our carbonized samples, also confirmed by the EDX analysis, breaks the symmetry of the carbon network, and these bands become active in the FTIR spectra [41,42,43].

#### 2.1.3. X-ray Diffraction

**Figure 3:**
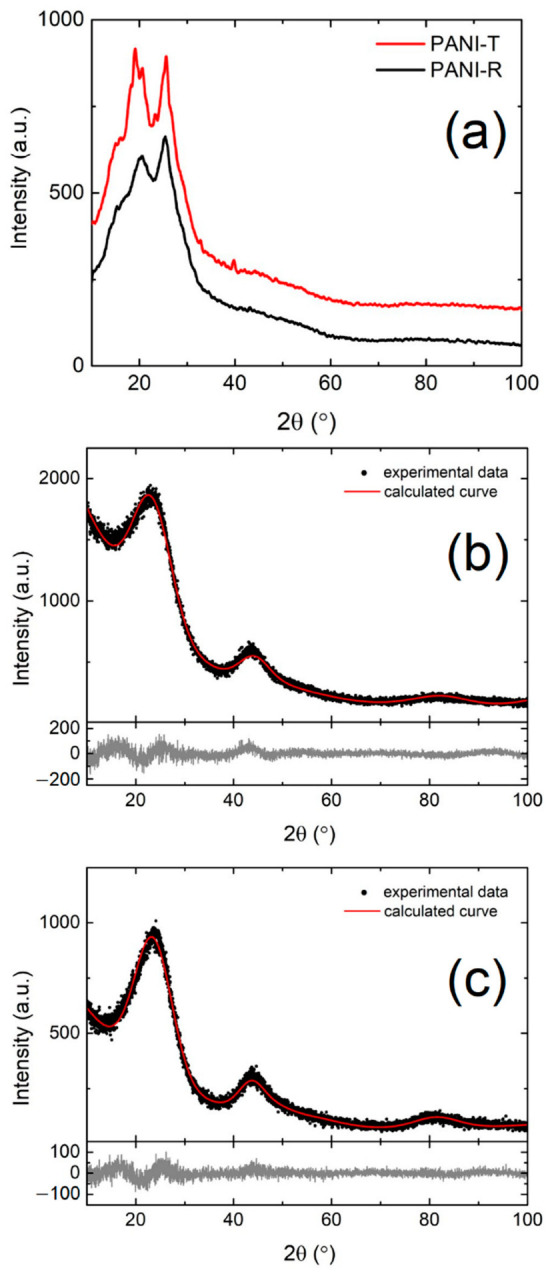
(**a**) XRD patterns of PANI-R and PANI-T; XRD experimental data and Rietveld refinement with residuals at the graphs bottom of (**b**) PANI-R-900 and (**c**) PANI-T-900.

The X-ray diffraction patterns from Figure 3 present the structural characterization of both polymers and the NCNs obtained after thermal treatment of polymers. In Figure 3a, the XRD analyses of PANI-R and PANI-T show two broad peaks at 2θ values of 20.2° and 25.5°, assigned to the parallel and perpendicular periodicity to the polyaniline chain’s direction, respectively [44,45], which indicates the amorphous phase of samples. Notably, the XRD scattering pattern of the PANI-T highlights better-defined peaks, indicating the formation of a more ordered structure compared to PANI-R. This may be due to the inter-chain hydrogen bonding or electrostatic interaction between adjacent polymer chains caused by differences in morphology and preparation method [44,46,47]. Based on these results, the morphology of the polymer materials can influence the crystallinity of the sample, as reported in the literature [45]. Moreover, Figure 3b,c shows the XRD profiles of PANI-R and PANI-T samples after the thermal treatment performed in an N_2_ atmosphere at a temperature of 900 °C (PANI-R-900 and PANI-T-900, respectively). The carbonization of PANI-R and PANI-T is highlighted by the presence of two broad peaks corresponding to diffraction planes (002) and (101) of a disordered graphite phase [48]. To obtain quantitative information on the microstructural features, the Rietveld analysis was performed on experimental data using the Diffraction (MAUD) program [49]. For both samples, the crystalline coherence length and microstrain were obtained after Rietveld refinement of structural parameters. In sample PANI-R-900, the value of the crystallite size is 3.7 ± 0.6 nm, which is very similar to PANI-T-900, which was 3.8 ± 0.7 nm. Further, the microstrain has a slightly different value for PANI-R-900, 1.1 × 10^−1^ ± 2.4 × 10^−3^, in comparison with 9.6 × 10^−2^ ± 2.1 × 10^−3^ for PANI-T-900. These sensitive differences in microstructural parameters between the two samples also show that the nanofiber morphology of PANI-T, which has a better periodicity of polymer chains, favored the formation of a more ordered graphite phase than the sample PANI-R.

### 2.2. Performance of MFCs with NCN-Modified Anodes 

**Figure 4:**
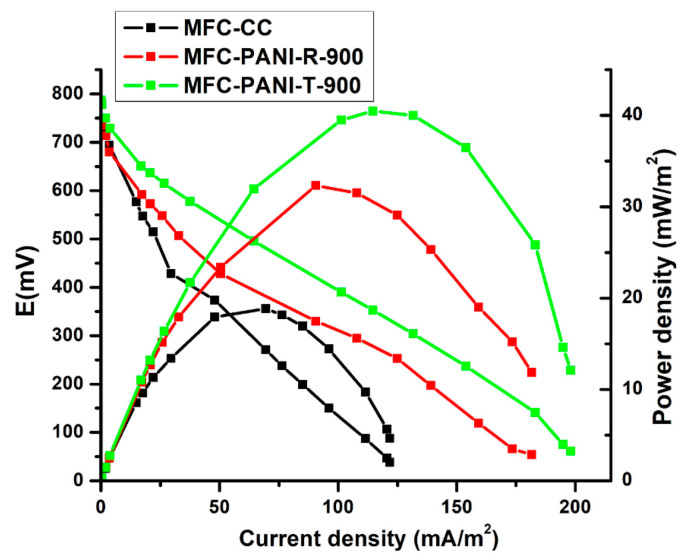
Polarization and power density curves of MFC with CC and PANI-R-900- and PANI-T-900-modified anodes.

In our study, 1 kΩ external resistance was used for anode biofilm inoculation. For the polarization measurements, the optimal resistance, defined as the external resistance for producing maximum power in MFCs, or a high external resistance (1 kΩ), which can facilitate a more compact structure of the biofilm, can be used [50,51]. As reported, it is important to have a mature anode biofilm with stable performance, and for an MFC with an H-type configuration, the operational voltage becomes stable 15 days after inoculation, or in less time if a mixed-culture anode is used [50]. In our case, the voltage became stable after 145 h for the MFCs using the PANI-T-900-modified anode and 200 h for MFCs using the PANI-R-900-modified anode. The polarization measurements confirm that both modifications of carbon cloth with PANI-R-900 and PANI-T-900 as MFC anodes showed an ability for electricity generation. The power density and polarization curves generated in MFCs are shown in Figure 4. The maximum power densities were affected by the NCN used for anode modification, being well recognized that the nature of modifier materials influences the performance of MFCs [7,8,9]. As can be seen in Figure 4, the maximum power density of 40.4 mW/m^2^ is obtained in the case of anode modification with NCNs with nanofibrillar morphology, PANI-T-900, compared with 32.3 mW/m^2^ obtained for anode modification with PANI-R-900 and 18.8 mW/m^2^ for MFC with carbon cloth (CC) anode. The average values (four replicas for each modification) of open circuit potential (OCP), power and current density and internal resistance for MFC, which used the same modifier for the MFC anode, PANI-R-900 and PANI-T-900, are summarized in Table 2. 

The polarization measurements show that the internal resistance of the cell followed the order: CC (1272 Ω) > PANI-R-900-modified CC anode (839 Ω) > PANI-T-900-modified CC anode (782 Ω) (see Table S1). According to the results obtained, the anode modification with PANI-T-900 is considered to be more beneficial for the improvement of MFC performance in terms of power generation. The nanofibrillar morphology of PANI-T-900, with both its larger specific surface area, given by the well-defined individual NCNs separated by microgaps and micropores, and higher conductivity, due to a better periodicity of polymer chains, which is also preserved in the carbonised samples, can facilitate better bacterial colonization and more efficient substrate transport [52]. Based on the NCN characterizations, the NCN with the nanofibrillar morphology provides a better organization of the carbon network, which implies a higher conductivity and a small content oxygen content in the carbon network that may improve the performance of the MFC [53,54]. Moreover, the fiber shapes can accelerate both direct and indirect electron transfer to optimize the MFC output [54]. As a consequence, the better performance of the MFC with the modified PANI-T-900 anode may be associated with the nanofibrillar morphology of NCNs.

### 2.3. Biofilm Characterization

The microbial community formed on the surface of the PANI-R-900- and PANI-T-900-modified CC anodes, as well as the microbial community of the initial wastewater, were characterized through metabarcoding techniques. The V_3_V_4_ region of the bacterial 16S rRNA gene from the total biofilm DNA samples was amplified and sequenced using NGS (Illumina, San Diego, CA, USA) technologies.

Results show that the operating conditions as well as the anode type determined an enrichment of select taxa from the initial wastewater. Regarding alpha-diversity metrics (Table 3), there was an increase in both Shannon entropy and Faith’s phylogenetic distance (Faith’s PD) from the initial wastewater to the anode biofilms. The PANI-R-900-modified anode had the highest Shannon diversity of all sample types, indicating the greatest variation in feature relative abundance. Faith’s PD increased significantly (*p* < 0.05) in the anode biofilms from the initial wastewater. Pielou’s evenness values did not differ greatly, although a slight decrease was observed in the case of the PANI-T-900-modified anodes, indicating a larger variation within more closely related taxa when compared to the PANI-R-900-modified anodes.

**Figure 5:**
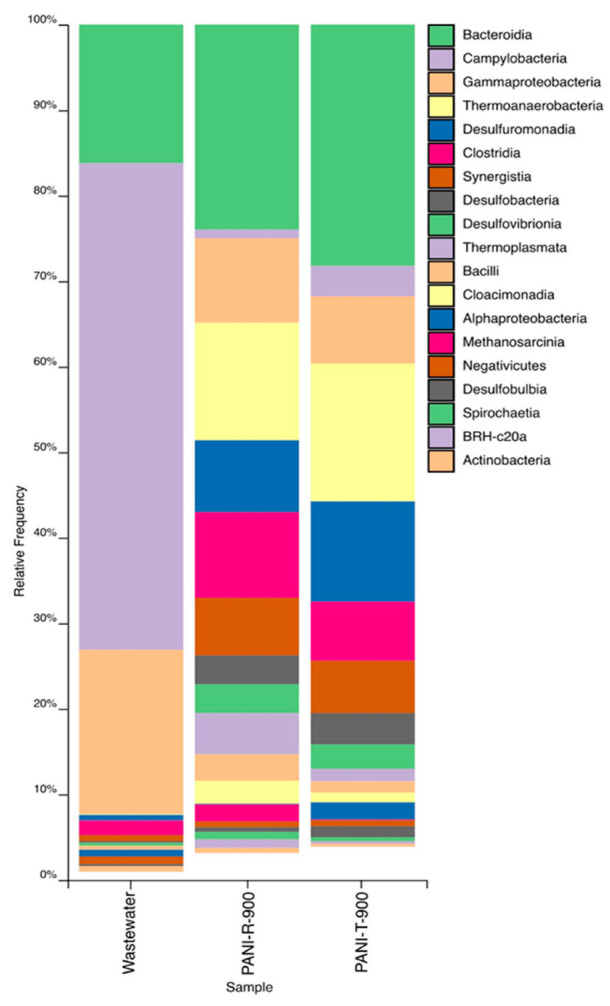
Barplot representation of most abundant classes across the three sample types. Relative frequencies represent the mean-ceiling of triplicate datasets.

Feature taxonomy at the class level underwent major changes when comparing the most abundant taxa from the initial wastewater to the anode biofilms (Figure 5). The initial wastewater had a community consisting of mainly Campylobacteria, of which the main constituents were *Arcobacter* sp. (22.9%), *Pseudoarcobacter* sp. (14.81%), and unclassified *Arcobacteraceae* (17.83%), the family being one of the most abundant in raw sewage and wastewater samples [55]. Other abundant classes were *Gammaproteobacteria* (19.19%) and *Bacteroidia* (16.22%), also commonly present in wastewater [56]. 

In the anode biofilms, *Campylobacteria* and *Gammaproteobacteria* relative abundance decreased greatly, while *Bacteroidia* abundance increased, more so in the case of PANI-T-modified anodes, 28.22% compared to 23.9% for PANI-R-900. The most enriched classes for both anode types were *Thermoanaerobacteria* (PANI-T-900—16.12%; PANI-R-900—13.73%), *Desulfuromonadia* (PANI-T-900—11.73%; PANI-R-900—8.42%), *Clostridia* (greater abundance in PANI-R, 9.99%, and 6.88% in PANI-T), *Synergistia*, *Desulfobacteria* and *Desulfovibrionia*. 

**Figure 6:**
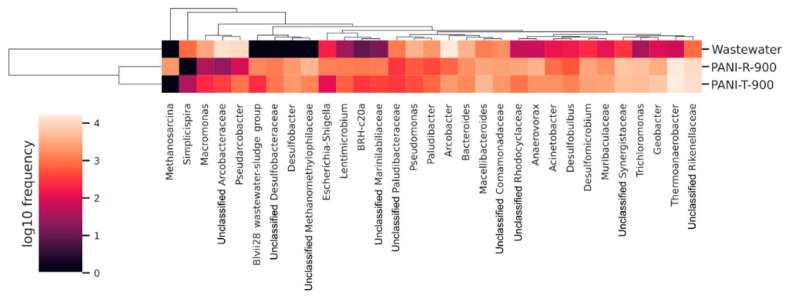
Heatmap representation of genera of at least 1% relative abundance, log normalized. Samples and features were clustered based on Bray-Curtis distances. Relative frequencies represent the mean-ceiling of triplicate datasets.

Taxa classified by the SILVA database as belonging to the *Thermoanaerobacteria* class are more commonly classified into *Clostridia*, the only genus present being *Thermoanaerobacter* (Figure 6). Species belonging to this genus have been observed as dominant in other MFC systems and also used in pure culture form for electrical current generation, colonizing the system anodes [57,58]. *Desulfuromonadia*, *Desulfobacteria* and *Desulfovibrionia* are members of the *Desulfobacterota* phylum, whose members are characterized by their dissimilatory sulphate reduction capability [59], a trait relevant to the bioremediation component of MFC functionality. *Synergistia* includes bacteria commonly associated with acetate metabolism [60], and its presence could be justified by the addition of the initial wastewater through acetate addition. Although the air present in the anodic chamber was not removed through special procedures, the main taxa forming the biofilm on the MFC anodes are obligate anaerobes, indicating that throughout the MFC operation period, oxygen was depleted, allowing the enrichment of these bacterial classes. Additionally, the greater abundance of anaerobic taxa in the biofilm of the PANI-T-900-modified CC anode could be due to the decreased oxygen content of the nanofibrillar structures.

MFC operation determined an enrichment of *Archaea*, from an abundance of 0.035% in the initial wastewater to 7.26% and 1.77% in the PANI-R- and PANI-T-modified CC anodes, respectively. The presence of *Archaea*, namely *Methanosarcinales* (class *Halobacterota*, 1.96% in PANI-R-900) and *Methanomassilicoccales* (class *Thermoplasmata*, 4.79%—PANI-R-900, 1.44%—PANI-T-900), a characteristic of anaerobic systems, could bring added value to the MFC system through methanogenesis from acetate [56].

At the genus and family levels (Figure 6), results show multiple taxa with high relative abundance, which are frequently associated with the ability for extracellular electron transfer (EET). The *Geobacter* genus, the type genus studied for its exoelectrogenic capabilities [61,62], had a relative abundance more than twofold greater in the case of the PANI-T-900-modified CC anode (6.62%) than the PANI-R-900-modified CC anode (3.05%), representing a possible justification for its better power generation. Furthermore, *Thermoanaerobacter* sp. (as mentioned previously) and *Macellibacteroides* [63] were more abundant in the PANI-T-900 CC biofilm, possibly contributing to MFC performance.

Other known EET-capable bacterial taxa enriched in the case of both anode modifiers used are *Trichloromonas* [64] and members of the *Rhodocyclaceae* and *Comamonadaceae* families [65].

## 3. Materials and Methods

### 3.1. Synthesis of Nitrogen-Containing Carbon Nanostructures

Direct carbonization of polyaniline (PANI) as an N-containing aromatic polymer with N covalently bonded in polymeric precursors is an efficient way to prepare N-containing carbon nanostructures, most frequently by preserving the morphology of the PANI precursor.

Two different synthesis methods were used to obtain different morphologies for PANI precursors. In the first method, polyaniline nanostructures were prepared by the chemical oxidative polymerization of an aniline monomer in acid media using ammonium persulfate as the oxidizing agent, following a modified procedure of an adapted method previously reported by Rezvani et al. [66]. In a typical reaction, 0.1 M aniline was dissolved in 0.1 M H_2_SO_4_ under stirring conditions at room temperature for 30 min. A total of 0.1 M ammonium persulfate was dissolved in 50 mL of 0.1 M H_2_SO_4_ and pre-cooled before adding to the monomer solution drop by drop. The polymerization solution was allowed to react overnight at room temperature. The resulting precipitate was collected and washed several times with deionized water and methanol. Finally, the product was dried at 60 °C overnight and indexed as PANI-R. The second procedure for the synthesis of polyaniline nanostructures was carried out using a template-free self-assembly method in an appropriate mixed solution of 0.4 M ethanol and 0.4 M acetic acid, as reported in reference [67]. Separately, 1.82 mL of aniline monomer was dissolved in 100 mL of the mixed solution, and 5.71 g of ammonium peroxydisulfate was dissolved in 100 mL of the mixed solution. The solutions of aniline and oxidant were cooled at a temperature between 0–5 °C for 30 min and then mixed rapidly into a beaker while being stirred vigorously for 30 s. The mixture was left to react overnight at ~0–5 °C, and then the product was filtered and washed with deionized water and methanol and finally dried at 50–60 °C in an oven overnight at ambient conditions. The final product was indexed as PANI-T.

NCNs were obtained after carbonization of PANI-R and PANI-T at 900 °C under nitrogen flow, with a 5 °C/min heating rate and 1 h holding time at 900 °C, followed by free cooling to room temperature under nitrogen flow. The NCN samples were indexed as PANI-R-900 and PANI-T-900, respectively.

### 3.2. Material Characterization

The morphological characteristics and elemental composition of PANI nanostructures and corresponding NCNs were analyzed using scanning electron microscopy (Tescan MAIA-3 field-emission electron microscope (TESCAN, Brno, Czech Republic), using a secondary electron detector and 5 kV accelerating voltage), coupled with an energy dispersive X-ray spectrometer (EDX QUANTAX 200 X-Flash 6/30, from Bruker, Billerica, MA, USA). Quantification of the EDX spectra was performed using the Kα lines by means of the Bruker ESPRIT Quant v2.3 software (Bruker, Billerica, MA, USA).

Fourier transform infrared (FTIR) spectroscopy analysis of the PANI nanostructure samples was performed by recording 128 scans in the range of 600–4000 cm^−1^, with a resolution of 4 cm^−1^ on a Varian 3100 Excalibur spectrophotometer (Varian, Palo Alto, CA, USA). The samples were analyzed by attenuated total reflection (ATR) using a Pike MIRacle (Ge crystal) accessory. The crystalline features of PANI nanostructures and corresponding NCNs were characterized by X-ray diffraction (XRD) using a Bruker D8 Discover diffractometer (CuK_α_ = 1.54 Å) in symmetric geometry. The data were collected in the interval of 2θ = 10–80° with an angular step of 0.02° at ambient conditions.

### 3.3. Microbial Fuel Cell Design and Operation

The double chamber is commonly used in laboratory research for the investigation of new substrates, electrode materials, membranes or types of microbial communities, and it is known for its low power generation because of its complex design and high internal resistance [7,68]. A double chamber MFC with an H-type configuration (Figure S6), made of glass and separated by a Nafion 117 Proton Exchange Membrane (PEM), was used to test the performance of the anode modified with NCNs. Anodes were made of non-wet proofed carbon cloth (CC) modified with NCNs and connected to the external circuits containing a 1 kΩ resistor using titanium wire and crocodile clips. For the anode modification, a suspension of NCNs (1 mg/mL) in ethanol aqueous solution (1:1 *v*/*v*) containing 0.5% Nafion was prepared. After homogenization in an ultrasonic bath for 30 min, the ink was sprayed onto the carbon cloth surface using an airbrush. The loading of NCNs on carbon cloth for all MFCs was ~1.1 mg/cm^2^. For Pt-containing cathodes, commercial Pt catalyst (40 wt% Pt/C, Alfa Aesar, Haverhill, MA, USA) ink mixed with a chemical binder (5% Nafion solution) was sprayed over carbon cloth. The anolyte consisted of municipal wastewater (S.C. Distributie Apa si Canalizare Magurele SRL, Ilfov Romania) supplemented with 1 g/L of acetate, and phosphate buffer (0.05 M, pH 7.0 ± 0.01) was used as the catholyte. The anode chamber was hermetically sealed, while the cathode chamber was purged with bubbling air. Cell voltages across the external resistor were recorded using a Pico Data Logger ADC-24 (Pico Technology, Cambridgeshire, UK) and a personal computer. For each modification, four identical MFCs with PANI-R-900- and PANI-T-900-modified CC anodes were operated simultaneously and compared with MFC using a CC anode as a controller. The polarization curves and power output were obtained by varying the external resistor from 1 MΩ to 40 Ω. The open cell potential (OCP) was measured after 120 min. At each resistance, MFC ran for at least 10 min to ensure that a stable power output had been achieved. Current (I) was calculated as I = V (cell voltage)/R (external resistance), and power (P) was calculated as P = I × V. Both current density and power density were calculated based on the surface area of the anode.

### 3.4. Biofilm Sample Collection and DNA Extraction. Sequence and Statistical Analysis

Anode biofilm and initial wastewater total DNA were isolated using the Dneasy PowerSoil Kit (#12888; Qiagen, Hilden, Germany) according to the manufacturer’s protocol, including the additional incubation step at 70 °C before vortexing samples. DNA, RNA, and protein quantification were done using a Qubit 4.0 and the dsDNA BR (#Q32850; Thermo Fisher Scientific, Waltham, MA, USA), RNA HS (#Q32852, Thermo Fisher Scientific), and Protein (#33211, Thermo Fisher Scientific) Assay Kits. Triplicate samples were selected for each anode type (PANI-R-900; PANI-T-900) and initial wastewater based on quality and DNA concentration. Sequencing was externalized towards Novogene (https://en.novogene.com/, accessed on 15 march 2022). Samples were amplified using universal primers for the V_3_-V_4_ region of the bacterial 16S rRNA gene (sequencing depth—100 k tags). The PCR products were selected by 2% agarose gel electrophoresis (Agilent 5400, Agilent, Santa Clara, CA, USA). Equal amounts of PCR product from each sample were pooled, end-repaired, A-tailed, and further ligated with Illumina adapters. Libraries were sequenced on a paired-end Illumina platform to generate 250 bp paired-end raw reads. Data analysis was performed in-house using the QIIME2 toolkit [69]. Sequencing data were denoised and quality-controlled using DADA2 [70]. SEPP [71] was used for the phylogenetic placement of ASVs into a reference tree based on the SILVA 128 database [72]. A classifier based on the SILVA 138 99% database was used, together with the q2-feature-classifier (classify-sklearn) plugin [73], for the taxonomical annotation of ASVs.

## 4. Conclusions

In this paper, polyaniline with different morphologies was used as a precursor for the synthesis of nitrogen-containing carbon nanostructures. The NCNs with different morphology were considered for anode modification, and the resulting MFC performance was studied and compared with MFC with carbon cloth anode as controls. The maximum power density of 40.4 mW/m^2^ was obtained in the case of anode modification with PANI-T-900, which has a nanofibrillar morphology, being 1.25 times higher when compared to the anode modified with PANI-R-900 and 2.15 times higher than the MFC using the CC anode. The internal resistance reduction also resulted in the improvement of power generation. MFC operation determined the enrichment of various taxa, resulting in community profiles that differed greatly from the initial wastewater. The dominant taxa in the anode biofilms were part of genera and families associated with extracellular electron transfer, such as *Geobacter*, *Thermoanaerobacter*, *Rhodocyclaceae*, and *Comamonadaceae*. The use of PANI-T-900-modified CC anodes resulted in greater relative abundances for members of *Geobacter* and *Thermoanaerobacter*, justifying the MFCs’ better power generation. The majority of taxa identified were anaerobic, while bacteria from the *Desulfobacterota* phylum indicated the occurrence of sulphate reduction. The presence of methanogenic *Archaea* in the biofilms could indicate the synthesis of methane, a possible value-added byproduct, throughout the process. Since the experimental results proved that both modifications with NCNs lead to a better performance of MFCs, polyaniline-derived nitrogen-containing carbon nanostructures can be considered a very good material for MFC anode modification, especially due to the simple and economical process that can allow the introduction of nitrogen functionality on the anode surface. Further research would be necessary to undertake in order to correlate the change in nitrogen functionality with NCN morphology.

## Figures and Tables

**Table 1:** Elemental composition of the materials from EDX.

Sample	C (at %)	N (at %)	O (at %)	S (at %)
PANI-R	73.34	10.99	12.92	2.74
PANI-T	82.08	8.08	6.20	3.64
PANI-R-900	89.95	6.49	3.56	NA
PANI-T-900	92.42	5.02	2.56	NA

NA—not available.

**Table 2:** Average values of open circuit potential (OCP), power and current density and internal resistance of MFC modified with PANI-R-900 and PANI-T-900 and MFC with CC anode.

MFC Index	OCP (mV)	P (mW/m^2^)	R_int_ (Ω)
MFC-CC	702.5 ± 24.5	15.2 ± 2.6	1437.6 ± 122.6
MFC-PANI-R-900	761.2 ± 28.7	30.6 ± 1.9	875.2 ± 52.9
MFC-PANI-T-900	789.7 ± 31.3	38.5 ± 1.9	808.7 ± 48.6

**Table 3:** Alpha diversity across sample types. Shannon entropy, Pielou’s evenness, and Faith’s phylogenetic distance were calculated using qiime2 core metrics phylogenetic.

Sample Type	Shannon	Faith’s Phylogenetic Distance	Pielou’s Evenness
Initial Wastewater	11.33 ± 0.28	322.26 ± 176.34	0.9407 ± 0.0027
PANI-R-900	12.16 ± 0.13	835.92 ± 86.04	0.9408 ± 0.0042
PANI-T-900	11.92 ± 0.12	811.30 ± 24.31	0.9294 ± 0.0049

## Data Availability

The raw sequencing data used in this study and associated metadata are openly available on the NCBI Sequence Read Archive under the accession numbers SRR20853640–SRR20853648 and BioSample accession numbers SAMN30164241–SAMN30164249, part of BioProject PRJNA866371.

## Supplementary Materials

The following supporting information can be downloaded at: www.mdpi.com/1422-0067/231/91/1230/s1.
