# Supplementary material for: Polyaniline-Derived Nitrogen-Containing Carbon Nanostructures with Different Morphologies as Anode Modifier in Microbial Fuel Cells
Source: Int J Mol Sci. 2022 Sep 23;23(19):11230. doi: 10.3390/ijms231911230 (PMC9569864; doi:10.3390/ijms231911230)
Supplement: Supplementary file 1 [file ijms-23-11230-s001.zip › ijms-1893579-supplementary.pdf]

Supplementary material

Article

Polyaniline-Derived Nitrogen-Containing Carbon Nanostructures with Different Morphologies as Anode Modifier in Microbial Fuel Cells

Irina Lascu [1], Claudiu Locovei [2,3], Corina Bradu [1], Cristina Gheorghiu [4], Ana Maria Tanase [1,*] and Anca Dumitru [2,*]

[1] Faculty of Biology, University of Bucharest, Splaiul Independenței 91–95, 050095 Bucharest, Romania
[2] Faculty of Physics, University of Bucharest, P.O. Box MG-11, 077125 Magurele, Romania
[3] National Institute of Materials Physics, Atomistilor 405A, 077125 Magurele, Romania
[4] Extreme Light Infrastructure-Nuclear Physics (ELI-NP), "Horia Hulubei" National Institute for R&D in Physics and Nuclear Engineering, P.O. Box MG-6, 077125 Magurele, Romania
* Correspondence: ana-maria.tanase@bio.unibuc.ro (A.M.T.); anka.dumitru@gmail.com (A.D.)

Table S1. Open circuit potential (OCP), power and current density and internal resistance of MFC modified with PANI-R-900 and PANI-T-900 and MFC with CC anode (four replicas for each modification).

**MFC**	**Anode modification**	**OCP(mV)**	**P(mW/m2)**	**Rint (ohm)**
MFC1	PANI-R-900	772	27.9	953
MFC2	PANI-R-900	797	32.3	839
MFC3	PANI-R-900	739	30.6	864
MFC4	PANI-R-900	737	31.4	845
**average**		**761.2**	**30.6**	**875.2**
**std**		**28.7**	**1.9**	**52.9**
MFC5	PANI-T-900	818	37.7	869
MFC6	PANI-T-900	807	39.8	825
MFC7	PANI-T-900	787	40.4	782
MFC8	PANI-T-900	747	36.1	759
**average**		**789.7**	**38.5**	**808.7**
**std**		**31.3**	**1.9**	**48.6**
MFC9	CC	719	15.0	1566
MFC10	CC	692	13.8	1441
MFC11	CC	726	18.8	1272
MFC12	CC	673	13.1	1472
**average**		702.5	**15.2**	**1437.6**
**std**		24.5	**2.6**	**122.6**

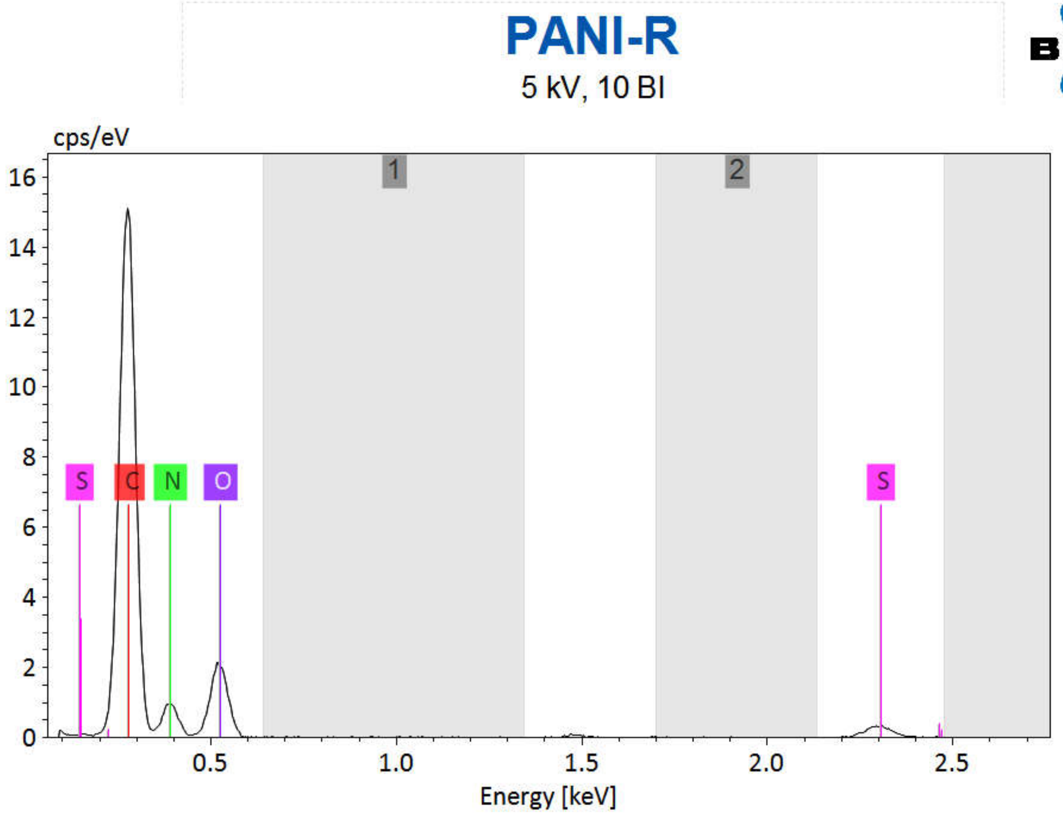

PANI-R_5kV_10BI

Element	At. No.	Netto	Mass [%]	Mass Norm. [%]	Atom [%]	abs. error [%] (1 sigma)	rel. error [%] (1 sigma)
Carbon	6	93929	66.26	66.26	73.34	1.94	2.94
Nitrogen	7	6400	11.57	11.57	10.99	0.46	4.00
Oxygen	8	14703	15.55	15.55	12.92	0.53	3.39
Sulfur	16	3838	6.62	6.62	2.74	0.19	2.88
		**Sum**	**100.00**	**100.00**	**100.00**		

Figure S1: Energy dispersive X-ray analysis (EDX) of PANI-R.

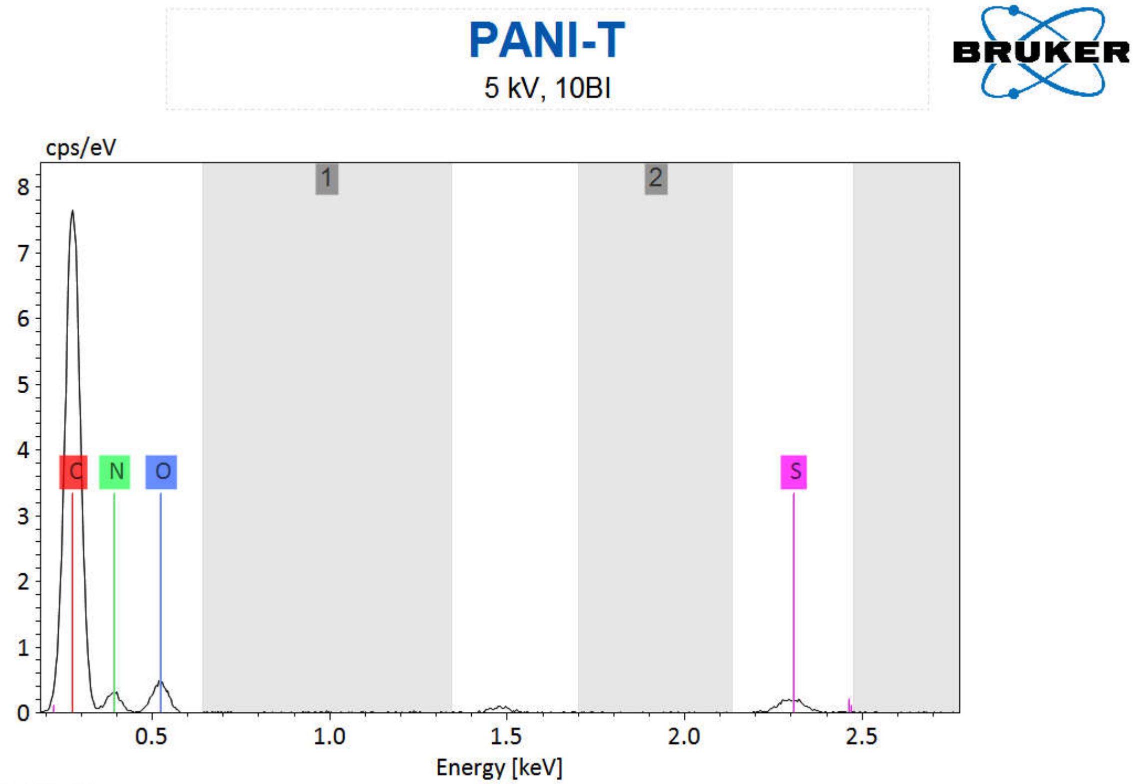

PANI-T_5kV_10BI

Element	At. No.	Netto	Mass [%]	Mass Norm. [%]	Atom [%]	abs. error [%] (1 sigma)	rel. error [%] (1 sigma)
Carbon	6	53938	74.98	74.98	82.08	2.24	2.99
Nitrogen	7	2248	8.61	8.61	8.08	0.36	4.21
Oxygen	8	3615	7.55	7.55	6.20	0.27	3.62
Sulfur	16	2686	8.87	8.87	3.64	0.25	2.78
		**Sum**	**100.00**	**100.00**	**100.00**		

Figure S2: Energy dispersive X-ray analysis (EDX) of PANI-T.

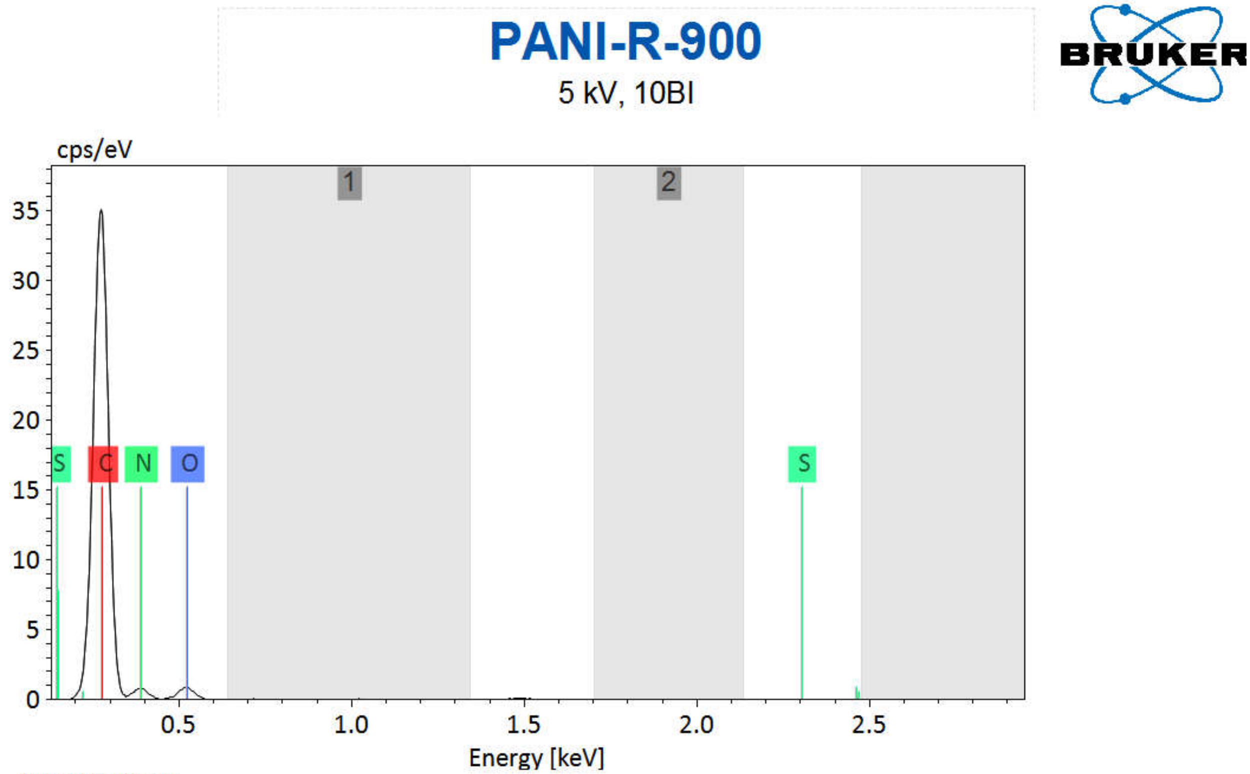

PANI-R-900_5kV_10BI

Element	At. No.	Netto	Mass [%]	Mass Norm. [%]	Atom [%]	abs. error [%] (1 sigma)	rel. error [%] (1 sigma)
Carbon	6	199885	87.96	87.96	89.95	2.33	2.65
Nitrogen	7	4773	7.40	7.40	6.49	0.32	4.32
Oxygen	8	5624	4.64	4.64	3.56	0.18	3.86
		Sum	100.00	100.00	100.00		

Figure S3: Energy dispersive X-ray analysis (EDX) of PANI-R-900.

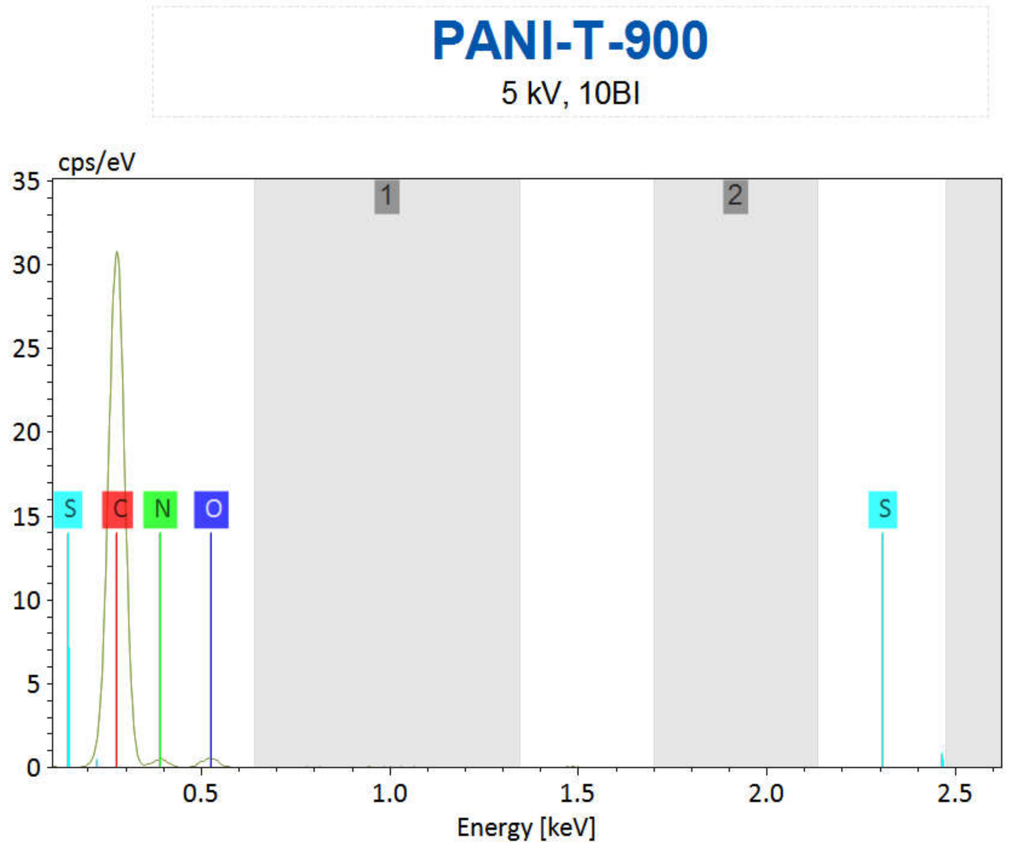

PANI-T-900_5kV_10BI

Element	At. No.	Netto	Mass [%]	Mass Norm. [%]	Atom [%]	abs. error [%] (1 sigma)	rel. error [%] (1 sigma)
Carbon	6	149125	90.89	90.89	92.42	2.41	2.65
Nitrogen	7	2542	5.75	5.75	5.02	0.26	4.45
Oxygen	8	2924	3.36	3.36	2.56	0.14	4.07
		Sum	100.00	100.00	100.00		

Figure S4: Energy dispersive X-ray analysis (EDX) of PANI-T-900.

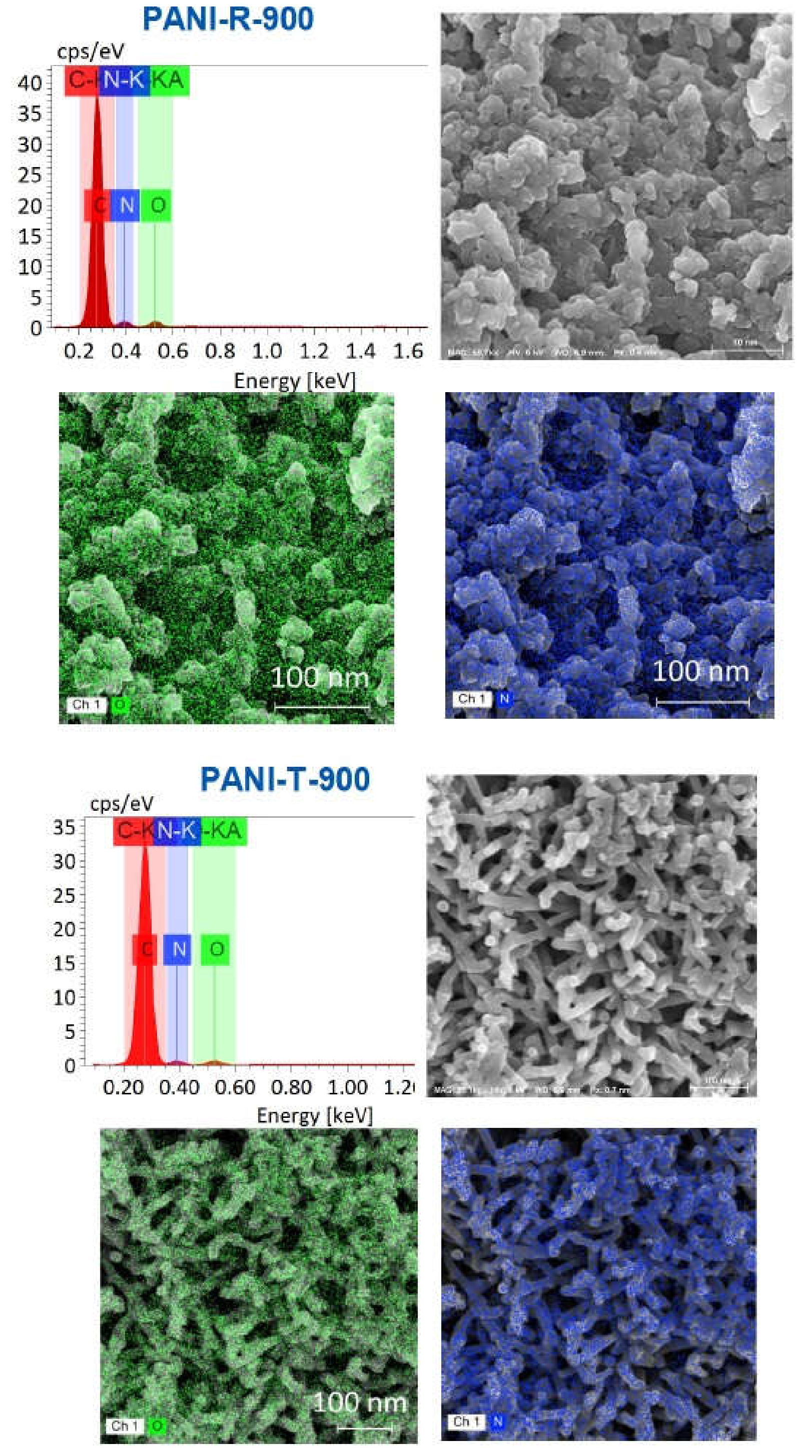

Figure S5. N and O EDX mapping of PANI-R-900 and PANI-T-900.

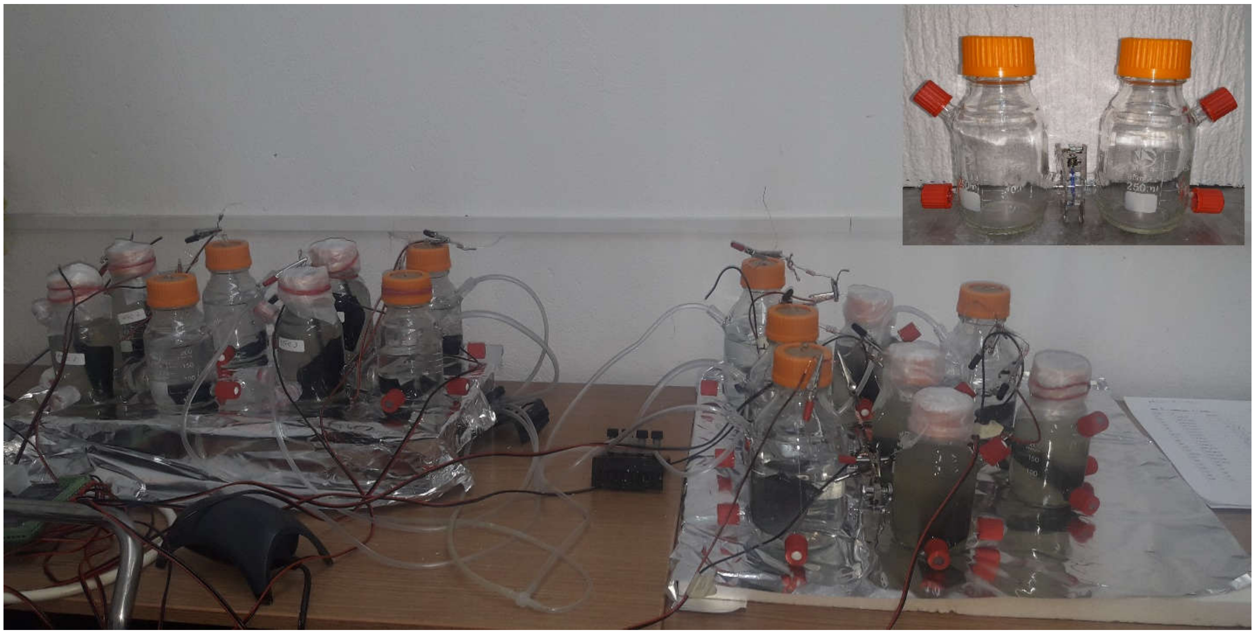

Figure S6. Setup and aspect of H-type configuration MFCs used in this paper.
